# The Akt Forkhead Box O Transcription Factor Axis Regulates Human Cytomegalovirus Replication

**DOI:** 10.1128/mbio.01042-22

**Published:** 2022-08-10

**Authors:** Hongbo Zhang, Anthony J. Domma, Felicia D. Goodrum, Nathaniel J. Moorman, Jeremy P. Kamil

**Affiliations:** a Department of Microbiology and Immunology, Louisiana State University Health Sciences Center– Shreveport, Shreveport, Louisiana, USA; b BIO5 Institute, University of Arizona, Tucson, Arizona, USA; c Department of Cellular and Molecular Medicine, University of Arizona, Tucson, Arizona, USA; d Department of Immunobiology, University of Arizona, Tucson, Arizona, USA; e Cancer Center, University of Arizona, Tucson, Arizona, USA; f Department of Microbiology and Immunology, Lineberger Comprehensive Cancer Center, University of North Carolina at Chapel Hill, Chapel Hill, North Carolina, USA; Princeton University

**Keywords:** AKT signaling, cytomegalovirus, herpesviruses, human herpesviruses, metabolism, protein kinases, stress response, transcription factors

## Abstract

The protein kinase Akt broadly impacts many cellular processes, including mRNA translation, metabolism, apoptosis, and stress responses. Inhibition of phosphatidylinositol 3-kinase (PI3K), a lipid kinase pivotal to Akt activation, triggers various herpesviruses to reactivate from latency. Hence, decreased Akt activity may promote lytic replication. Here, we show that Akt accumulates in an inactive form during human cytomegalovirus (HCMV) infection of permissive fibroblasts, as indicated by hypophosphorylation of sites that activate Akt, decreased phosphorylation of PRAS40, and pronounced nuclear localization of FoxO3a, a substrate that remains cytoplasmic when Akt is active. HCMV strongly activates mTORC1 during lytic infection, suggesting a potential mechanism for Akt inactivation, since mTORC1 negatively regulates PI3K. However, we were surprised to observe that constitutive Akt activity, provided by expression of Akt fused to a myristoylation signal (myr-Akt), caused a 1-log decrease in viral replication, accompanied by defects in viral DNA synthesis and late gene expression. These results indicated that Akt inactivation is required for efficient viral replication, prompting us to address which Akt substrates underpin this requirement. Interestingly, we found that short interfering RNA knockdown of FoxO3a, but not FoxO1, phenocopied the defects caused by myr-Akt, corroborating a role for FoxO3a. Accordingly, a chimeric FoxO3a-estrogen receptor fusion protein, in which nuclear localization is regulated by 4-hydroxytamoxifen instead of Akt, reversed the replication defects caused by myr-Akt. Collectively, our results reveal a role for FoxO transcription factors in HCMV lytic replication and argue that this single class of Akt substrates underpins the requirement for Akt inactivation during productive infection.

## INTRODUCTION

The serine/threonine protein kinase Akt regulates a broad range of cellular activities in response to signaling by receptor tyrosine kinases and G protein-coupled receptors (reviewed in reference 1). For instance, Akt enhances mRNA translation by phosphorylating PRAS40 and TSC2, which otherwise constrain mTORC1 activity (2, 3). Meanwhile, Akt promotes cell survival by phosphorylating the proapoptotic Bcl-2 family protein BAD (4). Akt directly phosphorylates various enzymes and proteins that regulate metabolism. For example, Akt phosphorylation of HK2 stimulates conversion of glucose to glucose-6-phosphate, while its phosphorylation of TXNIP inhibits endocytosis of glucose transporters from the plasma membrane (5). Although the positive influence of Akt on mRNA translation and inhibition of apoptosis might be expected to promote viral replication, a number of cytolytic viruses, such as measles virus and vesicular stomatitis virus, inactivate Akt during infection (6–8). Precisely how viruses benefit from shutting off Akt is unknown. However, some have hypothesized that certain viruses inactivate Akt to inhibit the translation of transcripts of interferon-stimulated genes (9).

Akt is activated during human cytomegalovirus (HCMV) entry into monocytes (10), and initial studies in lytically infected fibroblasts suggested that Akt is active or even upregulated throughout the lytic infection cycle (11–13). More recent findings, however, suggest that Akt becomes inactive during infection of permissive human fibroblasts (14, 15). Although cellular processes important for viral replication that ordinarily require Akt remain active during infection, HCMV expresses a protein called pUL38 that strongly activates mTORC1, by both TSC2-dependent and -independent mechanisms, which provides a mechanism to maintain high levels of mRNA translation regardless of Akt activity (16, 17). Yet, whether the virus benefits from maintaining Akt in an inactive state has remained unclear. One class of Akt substrates that may shed light on this unresolved matter is the Forkhead box O family transcription factors (FoxOs).

FoxOs coordinate transcriptional responses to metabolic and oxidative stress and strongly impact the expression of genes involved in immunity, apoptosis, cell differentiation, and cell cycle progression (18). Akt phosphorylates FoxO3a at Thr32, Ser253, and Ser315; phosphorylation at these sites recruits the binding of 14-3-3 proteins, which exclude FoxOs from the nucleus (19, 20). During growth factor withdrawal or oxidative stress, Akt activity ceases and FoxOs accumulate in the nucleus to transactivate stress response genes. We recently reported that FoxO3a plays roles in transactivating major immediate early (MIE) gene expression during HCMV reactivation from latency in myeloid cells (21, 22). Here, we investigate the significance of Akt regulation for lytic HCMV replication, specifically addressing its regulation of FoxO3a. We find that although Akt activity is maintained during the first few hours of infection, it is sharply downregulated by 12 h postinfection (hpi) of fibroblasts, and moreover, expression of a constitutively active Akt, myr-Akt, represses HCMV replication with corresponding reductions in viral gene expression and viral DNA synthesis. We further show that FoxO3a, but not FoxO1, is a key substrate of Akt important for HCMV replication. The nuclear localization of FoxO transcription factors is ordinarily negatively regulated by Akt (19, 23). However, by making use of a chimeric FoxO3a-estrogen receptor fusion protein, we are also able to demonstrate that forced localization of FoxO3a to the nucleus overcomes the defect in replication imposed by constitutive active Akt. Together, these findings establish FoxO transcription factors as crucial targets underlying HCMV regulation of the phosphatidylinositol 3-kinase (PI3K)/Akt pathway and reveal FoxO3a as a pivotal host transcription factor important for HCMV replication.

## RESULTS

### Akt is inactivated during HCMV infection of permissive cells.

**FIG 1:**
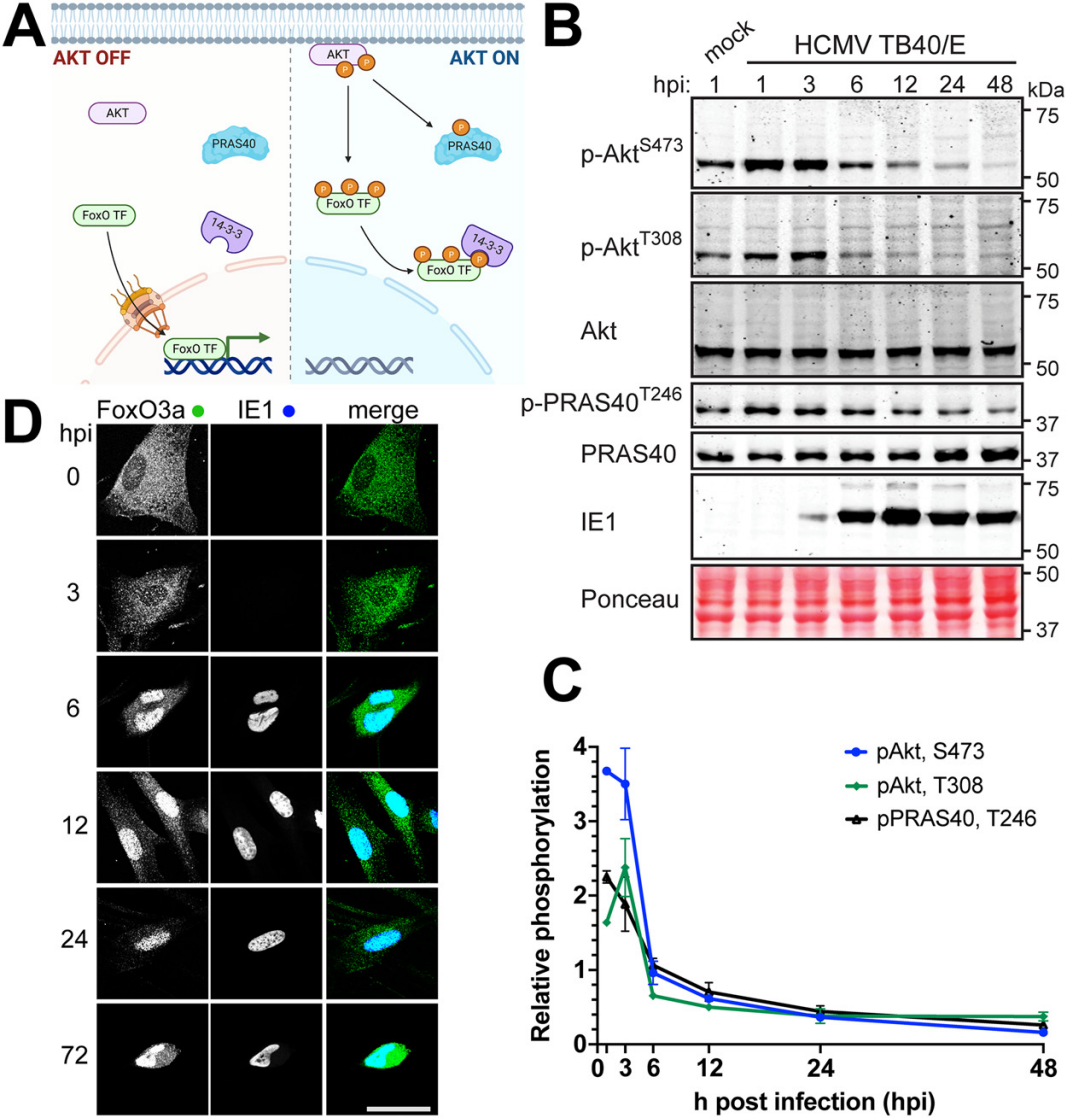
AKT is inactivated during HCMV infection. (A) Schematic denoting the influence of Akt activity on two key substrates, PRAS40 and FoxO transcription factors (FoxO TF). Left panel, when Akt is inactive, FoxO TF are able to enter the nucleus. Right panel, when Akt is recruited to membranes, such as during PI3K signaling, Akt is phosphorylated at activating residues Thr308 (T308) and Ser473 (S473). Once activated, Akt phosphorylates many downstream substrates, including PRAS40 and FoxO TF. Akt phosphorylation of FoxO TF recruits binding of 14-3-3 proteins, which sequester FoxO TF in the cytoplasm. (B) Fibroblasts were infected with HCMV strain TB40/E at an MOI of 2 or were mock infected (M) in the presence of 5% newborn calf serum; lysates were harvested at the indicated times postinfection (hours postinfection [hpi]) and analyzed by Western blotting for proteins immunoreactive to antibodies specific for the following antigens: p-Akt^S473^ and p-Akt^T308^, Akt phosphorylated at T308 or S473, respectively; Akt, total Akt; p-PRAS40^T246^, PRAS phosphorylated at T246; PRAS40, total PRAS40; IE1, the 72-kDa HCMV immediate early nuclear antigen. A section of nitrocellulose membrane was also subjected to Ponceau S staining (Ponceau) to assess total protein loading across lanes. (C) Fluorescent signals from far-red dye-labeled secondary antibodies used for Western blot detection of p-Akt^T308^, p-Akt^S473^, and p-PRAS40^T246^ were normalized to the detection signal for total Akt or total PRAS40, respectively. The mean signal is plotted relative to the baseline signal from a 1-h mock infection treatment, and data shown are the average of two independent biological replicates with error bars indicating standard deviations. (D) Fibroblasts were infected with HCMV strain TB40/E at an MOI of 1. The cells were fixed in 4% PFA at the indicated times postinfection and stained with antibodies specific for FoxO3a (green) or HCMV IE1 antigen (blue). Scale bar, 25 μm.

Akt, also known as protein kinase B, is negatively regulated during the productive (or “lytic”) HCMV replication cycle, and its kinase activity is suppressive to replication (14, 15). To further explore how HCMV regulates Akt activity, we infected telomerase-immortalized human fibroblasts with HCMV strain TB40/E at a multiplicity of infection (MOI) of 2 50% tissue culture infective doses (TCID_50_)/cell and used phosphospecific antibodies to monitor phosphorylation of Akt at two sites crucial for its kinase activity, Thr308 and Ser473, and of PRAS40 (proline-rich Akt substrate of 40 kDa) at Thr246, a canonical indicator of Akt kinase activity (24) (Fig. 1A and B). Thr308 is located within the T loop of the Akt catalytic domain, and its phosphorylation is essential for kinase activity, while Ser473 is found within a hydrophobic patch of amino acids at the C terminus; mTORC2 phosphorylation of this site is posited to recruit PDK1 to phosphorylate Thr308 (25–29). Phosphorylation results for Akt and PRAS40 were quantified from two independent biological replicates of the experiment shown in Fig. 1B that used signal intensity from far-red fluorescent dye-conjugated secondary antibodies (Fig. 1B, Supplemental Fig S1). Phosphorylation signals from each sample were normalized to the respective detection signals for total Akt or PRAS40, and the signal from cells harvested 1 h after mock infection was set to 1.0. For simplicity’s sake, we refer to this latter value, i.e., the signal from 1-h mock-infected cells, as the “baseline” (Fig. 1C).

Akt phosphorylation initially increased during infection (Fig. 1B and C; see Fig. S1 in the supplemental material). Ser473 phosphorylation signal peaked at 1 h postinfection (hpi), reaching 3.7-fold the baseline signal seen from 1-h mock-infected cells and remaining 3.5-fold above baseline at 3 hpi. Meanwhile, Akt Ser308 phosphorylation signals at 1 and 3 hpi were 1.6- and 2.4-fold above baseline, respectively, and PRAS40 Thr246 phosphorylation signals were 2.3- and 1.9-fold above baseline at those two initial points. Although our viral stocks did not contain serum, 5% newborn calf serum (NCS) was present in the medium used for both mock infection and HCMV infection; therefore, any signaling caused by serum was controlled for. Because HCMV-infected cells showed an increase above mock-infected cells, these results suggest that early events during HCMV entry activate Akt, which is consistent with results seen during viral entry into monocytes (10). By 6 hpi, however, detection signals for phosphorylation of Akt Ser473 and PRAS40 Thr246 returned to baseline, and Akt Ser308 phosphorylation signal fell to 65% of baseline (Fig. 1C). At 12 hpi, phosphorylation signals for Akt Ser473 and Ser308 were at 61% and 50% of baseline, respectively, and signal for PRAS40 phosphorylation at Thr246 was at 70%. By 24 hpi, Akt Ser473 and Ser308 phosphorylation signals were each just below 40% of baseline (37% and 38%, respectively) and PRAS40 Thr246 phosphorylation signal was down to 44% of the baseline value. Finally, at 48 hpi, Akt Ser473 and Ser308 phosphorylation signals were at 16% and 37% and PRAS40 Thr246 phosphorylation signal was at 26% of baseline. We note that phosphorylation of PRAS40 Thr246 decreased in lockstep with Akt phosphorylation status at Ser473 and Thr308, while the total abundance of PRAS40 and Akt held roughly constant. Nevertheless, our quantification procedure normalized the detection of each phosphoepitope to the signal for detection of the cognate protein, be it Akt or PRAS40. Overall, our results suggest that decreased Akt phosphorylation at Thr308 and Ser473 coincided with a decrease in phosphorylation of a canonical Akt substrate, PRAS40 (Fig. 1B and C).

10.1128/mbio.01042-22.3 FIG S1 LI-COR quantification of Western blot results from Fig. 1B and C. Replicates 1 and 2 were used to produce the graph in Fig. 1C. Download FIG S1, TIF file, 2.8 MB. Copyright © 2022 Zhang et al. 2022 Zhang et al. https://creativecommons.org/licenses/by/4.0/ This content is distributed under the terms of the Creative Commons Attribution 4.0 International license.

To provide an orthogonal phenotypic indicator for inactivation of Akt, we monitored the nuclear localization of the Forkhead box O family transcription factor FoxO3a by indirect immunofluorescence staining. Because Akt negatively regulates the nuclear localization of FoxO family transcription factors (FoxO TF), the nuclear accumulation of these transcription factors argues that Akt activity is deficient (Fig. 1A) (19, 23, 30). Although FoxO3a signal was detected predominantly within the cytoplasm at 0 hpi and 3 hpi, the protein localized to the nucleus by 6 hpi and remained there throughout the remainder of the 72-h infection time course (Fig. 1D). In contrast, a mock infection time course carried out in parallel showed little to no nuclear accumulation of FoxO3a, and nuclear FoxO3a signal did not increase over time (Fig. S2). From these experiments, we concluded that HCMV infection of permissive human fibroblasts is associated with accumulation of Akt in an inactive form, in agreement with previous observations (14, 15).

**FIG 2:**
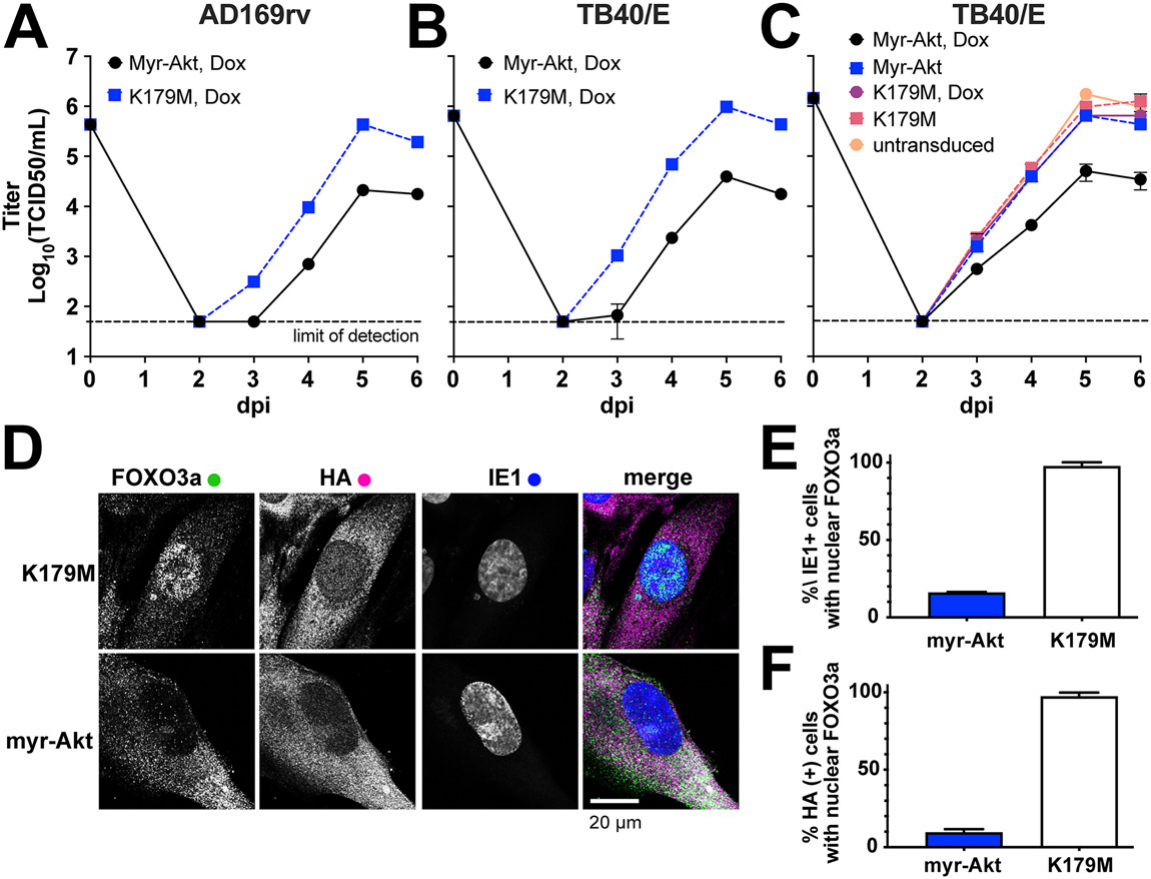
Constitutive AKT activity causes a viral replication defect. (A to C) Fibroblasts carrying doxycycline-inducible (tet-on) expression cassettes for either myristoylated Akt1 (myr-Akt) or a kinase-dead control (K179M) were induced for 24 h using 100 ng/mL doxycycline hyclate (Dox) and subsequently infected at an MOI of 1 with either HCMV strain AD169rv (A) or TB40/E (B and C). Supernatants were harvested at the indicated times postinfection (days postinfection [dpi]) and measured for the infectious titer by the TCID_50_ assay. (C) Results of an independent biological replicate of the infection experiment described in the legend for panel B but with additional controls: untransduced fibroblasts and tet-on myr-Akt and K179M fibroblasts in the absence of the transgene inducing agent (Dox). (D) Representative confocal microscope images of formalin-fixed fibroblasts at 24 h postinfection (MOI, 1; TB40/E) (see Fig. S1 in the supplemental material for a full time course from 0 to 72 h postinfection). (E and F) At least 30 cells per condition were scored at 72 hpi for the effect of HCMV infection (E) or myr-Akt or myr-Akt K179M expression (F) on nuclear localization of endogenous FoxO3a, as indicated on the *y* axis. HCMV infection was scored by detection of viral IE1 nuclear antigen in indirect immunofluorescent staining. Similarly, detection of HA epitope staining was used to score cells positive for expression of myr-Akt or K179M (the myr-Akt transgene carries an HA tag).

10.1128/mbio.01042-22.4 FIG S2 Full image time course series of the results shown in Fig. 1D and 2C. Confocal immunofluorescent staining data for the complete set of time points and controls related to the results shown in Fig. 1D and 2C are shown. Download FIG S2, PDF file, 0.9 MB. Copyright © 2022 Zhang et al. 2022 Zhang et al. https://creativecommons.org/licenses/by/4.0/ This content is distributed under the terms of the Creative Commons Attribution 4.0 International license.

### Constitutive Akt activity inhibits HCMV replication.

To address whether dampening of Akt kinase activity is required for efficient HCMV replication, we made use of a constitutively active form of Akt, myr-Akt, which carries a retroviral myristoylation signal at its N terminus such that localization to membranes, the rate-limiting step in Akt activation, is no longer regulated by PI3K. Making use of permissive human fibroblast cells stably transduced with “tet-on” expression cassettes that enable myr-Akt expression to be induced with doxycycline (Dox), we expressed either myristoylated Akt1 (myr-Akt) or a “kinase-dead” K179M myr-Akt control (K179M) for 24 h prior to infection at an MOI of 1 with either of two different HCMV strains, AD169rv and TB40/E. This system allowed us to ask whether the presence of constitutive Akt activity would impact the production of infectious particles over a 6-day time course. The two HCMV strains each exhibited a roughly 10-fold replication defect in cells expressing myr-Akt but not the kinase-dead control (Fig. 2A and B). Moreover, a viral replication defect was not seen in the absence of the transgene inducing agent (doxycycline) nor in fibroblasts that had not been transduced with a lentivirus vector encoding the tet-on Akt expression cassettes, which argues against causes other than constitutive Akt kinase activity (Fig. 2C).

As expected, infected cells expressing kinase-dead K179M myr-Akt showed robust nuclear localization of FoxO3a at 72 hpi, while cells expressing functional myr-Akt failed to show FoxO3a localization to the nucleus, consistent with the established role of Akt kinase activity in promoting nuclear export of FoxO transcription factors (19) (Fig. 2D to F). Results for the entire 0- to 72-h infection time course show that FoxO3a localized to the nucleus from 6 hpi through the end of the experiment in cells expressing kinase-dead myr-Akt, while myr-Akt-expressing cells failed to show FoxO3a nuclear localization at any time point, despite the fact that the viral antigen IE1 was detected in nuclei of both myr-Akt and K179M settings from 6 hpi on. From these results, we conclude that the presence of constitutively active myr-Akt during infection causes a substantial viral replication defect.

### Constitutively active Akt causes defects in viral gene expression and viral DNA synthesis.

**FIG 3:**
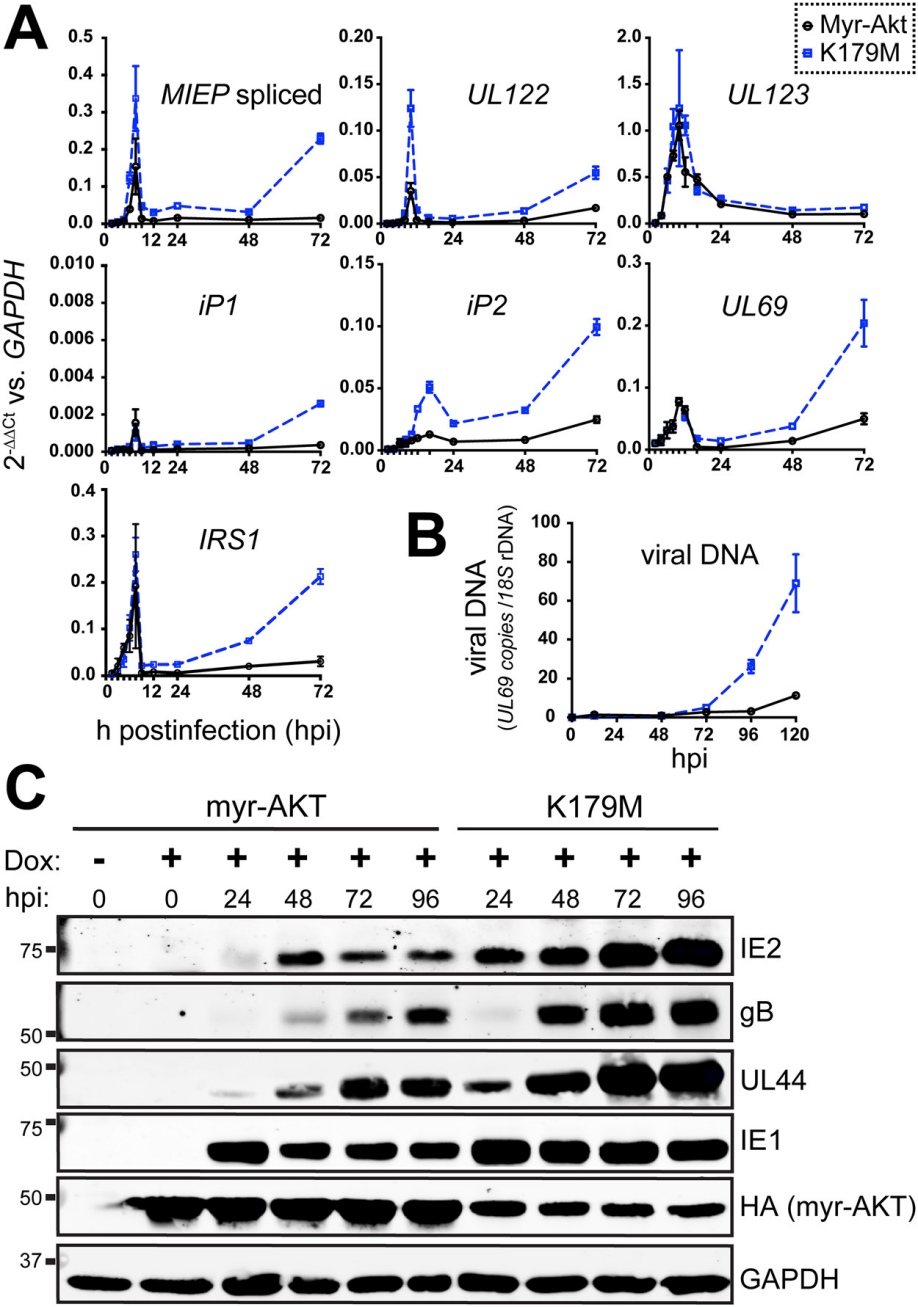
Constitutive Akt activity causes defects in viral gene expression and viral DNA synthesis. (A) Fibroblasts were induced for expression of either myr-Akt or myr-Akt-K179M (kinase dead) for 24 h and then were infected with HCMV strain TB40/E at an MOI of 1, and total RNA samples were isolated from either myr-Akt or K179M settings at 2, 4, 6, 8, 10, 12, 16, 24, 48, and 72 hpi, including an on-column DNase I digestion step. RNA samples were reverse transcribed into cDNA and assayed by qPCR for the abundance of the indicated viral transcripts relative to levels of cellular GAPDH mRNA. (B) Total DNA was isolated at the indicated times postinfection (hpi) from cells infected exactly as described for panel A, and copies of viral DNA were enumerated using quantitative PCR for the HCMV *UL69* gene normalized to copies of cellular 18S rDNA loci. (C) Infections were set up as described for panel A. Cell lysates collected at the indicated times postinfection were assayed by Western blotting for the expression of the indicated viral proteins, as well as for the expression of myr-Akt (HA tag) or GAPDH, as a loading control.

To better understand the nature of the viral replication defect that occurs during expression of constitutively active Akt, we next analyzed a selected set of viral RNA transcripts and proteins during HCMV strain TB40/E infection of fibroblasts (MOI, 1) expressing myr-Akt or a kinase-dead control. Reverse transcriptase quantitative PCR (RT-qPCR) results indicated that *UL123* (IE1) mRNA levels accumulated indistinguishably in myr-Akt and kinase-dead myr-Akt control settings (Fig. 3A). However, *UL122* transcripts, which encode the 86-kDa viral MIE transactivator protein IE2, accumulated to decreased levels during infection of myr-Akt-expressing cells but not during infection of comparator cells expressing a kinase-dead myr-Akt (K179M). A similar pattern was seen for the total levels of spliced transcripts originating from the major immediate early promoter (MIEP). Notably, our previous results from hematopoietic cell infection models indicate that FoxO3a transactivates promoters within intron A of the canonical MIE locus, contributing to the accumulation of IE2 (*UL122*) mRNA and protein (21, 22); these promoters, iP1 and iP2, were also previously found to contribute to IE2 expression during lytic infection of fibroblasts (31). The intronic promoters, particularly iP2, showed decreased activity in the presence of myr-Akt (Fig. 3A). Likewise, mRNAs for two other IE genes, *UL69* and *IRS1*, showed reduced accumulation during infection of cells expressing enzymatically competent myr-Akt relative to that of the K179M mutant control setting.

Defects in the expression of *UL122* (IE2) but not *UL123* (IE1) often relate to the late phase of IE2 expression, which unlike IE1 is sensitive to defects in viral DNA synthesis (32–36). Therefore, we examined whether viral DNA synthesis was affected during myr-Akt expression. Indeed, myr-Akt-expressing cells showed 6.1-fold reduced levels of viral DNA at 120 hpi relative to that of the K179M comparator condition (Fig. 3C). The observed decreases in *UL122* (IE2) mRNA expression and viral DNA synthesis were, as expected, accompanied by decreased protein levels for IE2, the late protein gB, and the early-late protein UL44 (Fig. 3A). In contrast, IE1 showed a less striking decrease in its protein expression level, and its RNA levels appeared to be unaffected by myr-Akt expression (Fig. 3A and B). Finally, although the “kinase-functional” myr-Akt protein accumulated to higher levels than its K179M kinase-dead counterpart, this was expected given that constitutive Akt kinase activity enhances mRNA translation capacity (reviewed in reference 37). Overall, these experiments indicate that constitutive Akt activity leads to defective accumulation of viral mRNAs and proteins and inefficient viral DNA synthesis.

### siRNA knockdown of FoxO3a causes viral replication defects.

Akt phosphorylates forkhead box O transcription factors (FoxOs) to prevent their nuclear localization (19, 23, 38), and our previous studies in HCMV latency models found that FoxOs promote reactivation. These observations led us to hypothesize that FoxO transcription factors play roles in lytic cycle progression. If so, the failure of FoxOs to reach the nucleus might contribute to the HCMV lytic cycle defects that we observed during expression of constitutively active myr-Akt. To address this possibility, we first turned to short interfering RNA (siRNA) to knock down the expression of either FoxO1 or FoxO3a, two Forkhead box O family transcription factors that are expressed in fibroblasts (39). Twenty-four hours after transfecting siRNA into fibroblasts, we infected the cells with HCMV strain TB40/E at an MOI of 1 TCID_50_/cell and in parallel experiments evaluated viral replication kinetics, viral DNA synthesis, and viral protein expression.

**FIG 4:**
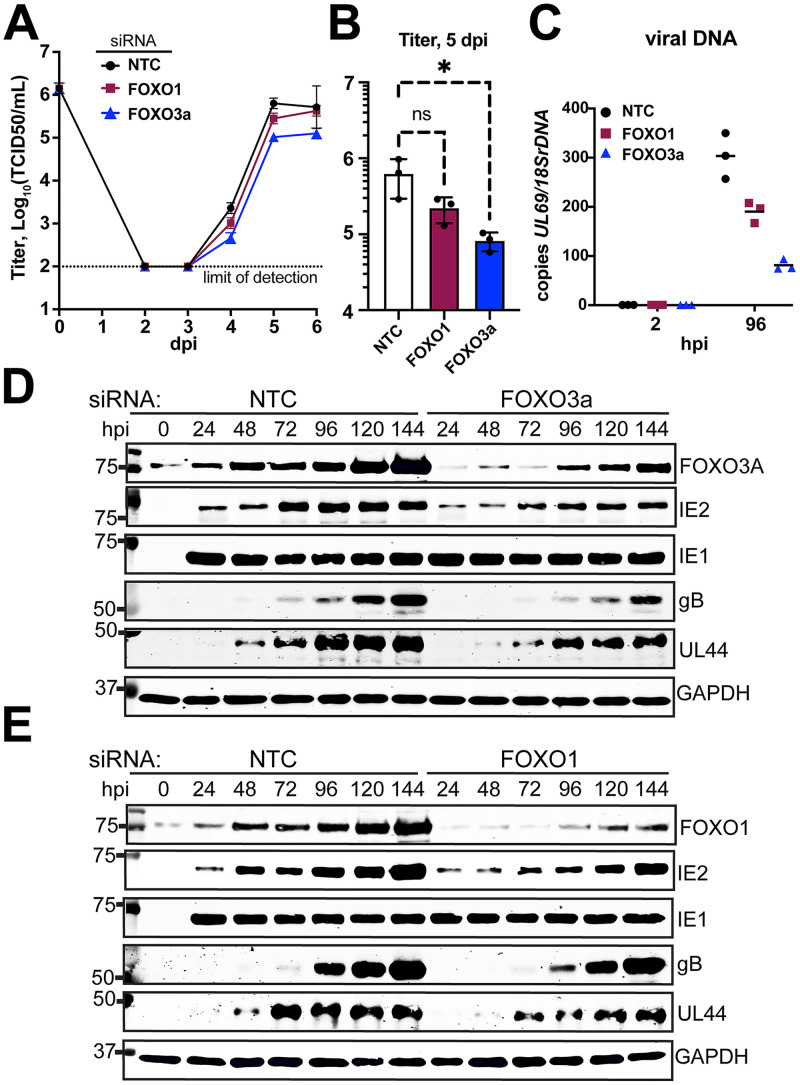
siRNA knockdown of FoxO3a causes viral replication defects. siRNA complexes targeting FoxO1, FoxO3a, or both FoxO1 and FoxO3a were reverse transfected into fibroblasts 24 h prior to infection with HCMV strain TB40/E at an MOI of 1. (A) Supernatants were harvested at the indicated times postinfection (days postinfection [dpi]). (B) Viral yield at day 5 postinfection for 3 independent biological replicates of the experiment described for panel A. An asterisk indicates a *P* value of 0.0276 using one-way analysis of variance (ANOVA) with Dunnett’s posttest to compare the mean of the results of each siRNA treatment condition to that of the nontargeting control (NTC) setting. (C) Viral DNA copies were enumerated by qPCR using a primer pair specific for HCMV *UL69* and are shown normalized to copies of cellular 18S rDNA loci. Results are shown for samples collected at 2 h postinfection (hpi) and 96 hpi to accurately indicate input levels of viral genomes against replicated viral DNA. (D and E) Protein lysates were obtained at the indicated times postinfection and analyzed by Western blotting to validate knockdown of FoxO3a or FoxO1, respectively, and to assess for effects on viral protein expression. Cellular GAPDH protein was detected as a loading control. NTC, nontargeting control siRNA.

Knockdown of FoxO3a caused a statistically significant roughly 1-log replication defect in the yield of infectious viral particles at 5 days postinfection (dpi), compared to that of the nontargeting (NT) siRNA control condition (*P* = 0.0276) (Fig. 4A and B). The average 5-dpi defect we observed during FoxO3a siRNA treatment was 7.5-fold (range, 5.0-fold to 11.2- fold). Silencing of FoxO1 caused a smaller replication defect, on average 2.8-fold at 5 dpi (range, 2.2-fold to 3.8-fold), which did not appear to be statistically significant. These viral replication defects were accompanied by inefficient viral DNA synthesis. At 96 hpi, cells silenced for FoxO3a showed a 3-fold reduction in viral DNA accumulation while FoxO1-silenced cells accumulated viral DNA at approximately two-thirds the levels of NT control settings (Fig. 4C and D).

Turning to protein expression, we were intrigued to observe that FoxO3a and FoxO1 protein levels were strongly upregulated during HCMV infection. Normalizing fluorescent detection signals for each FoxO transcription factor to those for GAPDH (glyceraldehyde-3-phosphate dehydrogenase) allows us to estimate that FoxO1 and FoxO3a were 4.4-fold and 3.0-fold more abundant at 120 hpi, respectively, than in uninfected cells (Fig. 4D and E; Fig. S3). The siRNA treatments kept FoxO protein levels below those seen in NT control-treated infections; quantification of secondary antibody fluorophore signals for detection of FoxO1 and FoxO3a at 120 hpi in the siRNA treatment settings indicated that the siRNA treatments reduced their expression by 60% relative to that at the equivalent time point in the NT control setting (Fig. 4D and E; Fig. S3). However, given that knockdown was incomplete, it is likely that more complete silencing of FoxO expression would lead to greater defects in viral replication and viral DNA synthesis.

10.1128/mbio.01042-22.5 FIG S3 LI-COR quantification of Western blot results from Fig. 4D and E. Download FIG S3, PDF file, 2.1 MB. Copyright © 2022 Zhang et al. 2022 Zhang et al. https://creativecommons.org/licenses/by/4.0/ This content is distributed under the terms of the Creative Commons Attribution 4.0 International license.

In agreement with the viral replication and DNA synthesis defects being greater during silencing of FoxO3a than FoxO1, FoxO3a-silenced cells showed poorer expression of glycoprotein B (gB), a late gene product (Fig. 4D and E). Accumulation of the viral DNA polymerase accessory factor UL44, which exhibits both early and late expression kinetics due to independently regulated transcription start sites in its promoter (40, 41), also showed a more severe decrease than that in the NT control setting in FoxO3a-silenced settings. At 120 hpi, GAPDH-normalized detection signals for gB and UL44 in FoxO3a siRNA-treated cells were decreased by 54% and 37% relative to that of NT control infections, respectively. Reduced expression of IE2 was also observed during FoxO3a silencing across all time points. The GAPDH-normalized IE2 signal was reduced by 57% relative to that of the NT control at 24 hpi, by 46% at 96 hpi, and by 43% at 120 hpi (Fig. 4D; Fig. S3). Although FoxO1-silenced cells likewise showed delayed accumulation of UL44 protein, as well as lower levels of IE2 than that of the NT control setting, these defects were more subtle than what we observed for FoxO3a (Fig. 4E; Fig. S3). From these experiments, we conclude that silencing of FoxO transcription factors can cause viral DNA synthesis and viral protein expression defects and that these appear to be more pronounced when FoxO3a is silenced.

### Forced nuclear localization of FoxO3a reverses viral replication defects caused by expression of constitutive Akt.

**FIG 5:**
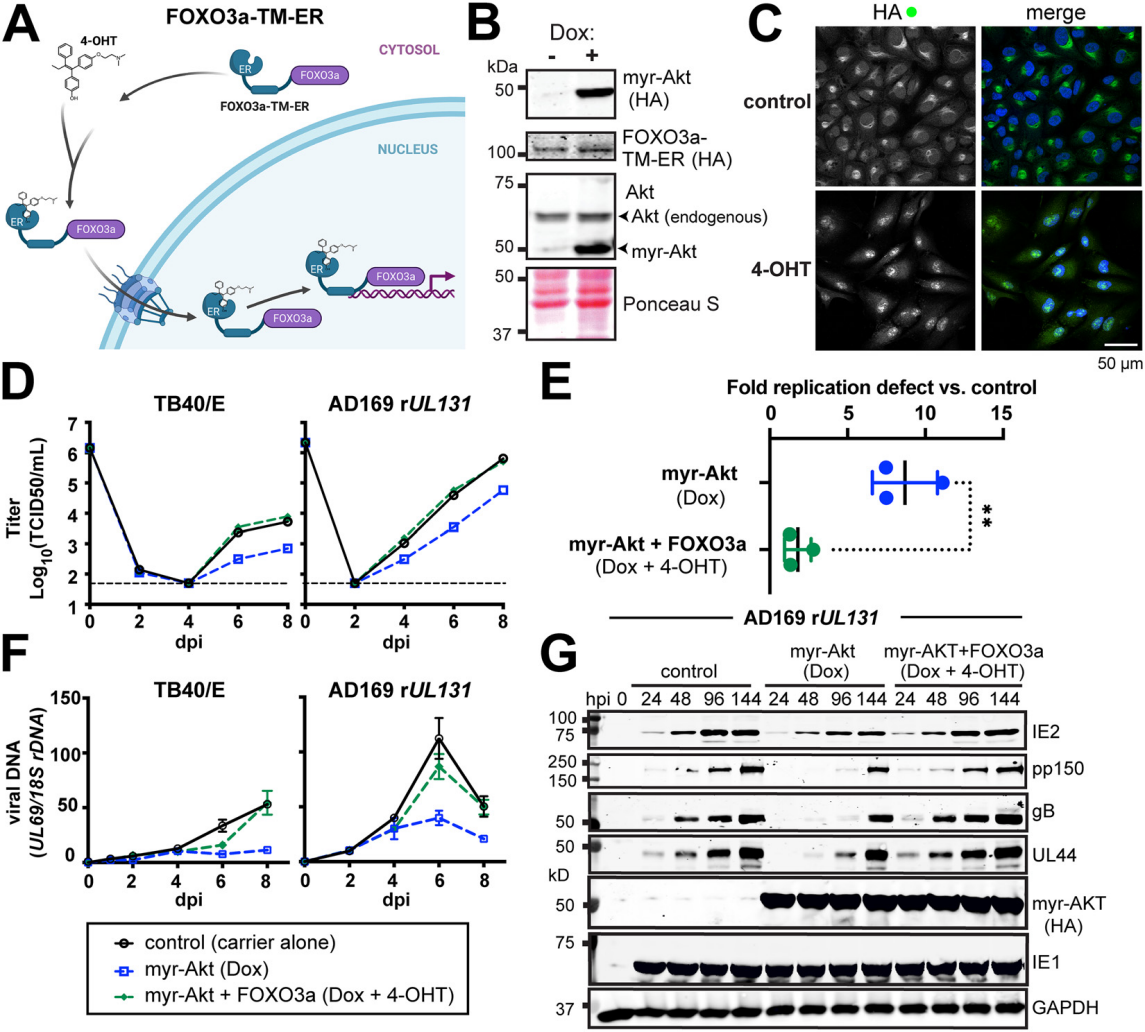
Hormone-regulated chimeric FoxO3a activity reverses Akt-dependent viral replication defects. (A) Cartoon depiction (created with BioRender) of chimeric FoxO3a “triple Akt phosphoacceptor site mutant” (TM), FoxO3a-TM-ER, in which FoxO3a-TM is fused to a mutant mouse estrogen receptor (ER) in which the antiestrogen 4-hydroxytamoxifen (4-OHT) activates its nuclear localization. Therefore, the FoxO3a-TM-ER protein allows FoxO3a activity to be decoupled from Akt regulation. (B) Western blot analysis of ARPE-19 epithelial cells carrying a doxycycline-inducible myr-Akt expression cassette (HA tagged) and constitutively expressing FoxO3a-TM-ER, also HA tagged. (C) Confocal immunofluorescence microscopy was used to validate 4-OHT-induced FoxO3a-TM-ER nuclear localization upon addition of 1 μM 4-OHT for 1 h. HA staining was used to detect the FoxO3a-TM-ER fusion protein; Hoechst 33342 signal is shown to counterstain nuclei in the merged image. (D) One-step viral replication kinetics at an MOI of 2 for HCMV strains TB40/E and AD169 repaired for *UL131* (AD169 r*UL131*) in ARPE-19 cells induced for myr-Akt expression (100 ng/mL doxycycline [Dox]) and with or without 4-OHT treatment (1 μM) to induce nuclear localization of FoxO3a-TM-ER, as indicated. (E) Results for viral replication yield defect at day 8 postinfection from 3 independent biological replicates of strain AD169 r*UL131* in ARPE-19 cells; the double asterisk indicates a *P* value of 0.0062 by an unpaired, two-tailed *t* test. (F) Viral DNA synthesis results for the same time points shown in panel D, comparing qPCR-detected copies of the HCMV *UL69* gene normalized to detection of cellular 18S rDNA copies. Note, the legend shown below panel F applies to panels D and F. dpi, days postinfection. (G) Western blot analysis of viral protein expression following AD169 r*UL131* infection (MOI, 2 TCID_50_/mL) in ARPE-19 cells in the presence or absence of Dox and/or 4-OHT treatments, as described above.

Our siRNA results suggested a more pronounced requirement for FoxO3a than FoxO1 during HCMV lytic infection (Fig. 4), echoing the role we found for FoxO3a in HCMV reactivation from latency in the THP-1 model and in CD34^+^ human progenitor cells (HPCs) (21, 22). Therefore, we next sought to interrogate the specific contribution of FoxO3a to the Akt-dependent growth defect we observed during lytic infection. Prior studies have made use of a chimeric FoxO3a estrogen receptor fusion protein, dubbed FoxO3a-TM-ER, to identify FoxO3a-regulated cellular genes. In this approach, FoxO3a is fused to the hormone binding domain of a mutant murine estrogen receptor (ER) carrying a G525R substitution. The G525R mutant has 1,000-fold reduced affinity for estrogen and is instead regulated by 4-hydroxytamoxifen (4-OHT), a synthetic antiestrogen (42, 43). Further, all three Akt phosphoacceptor sites on FoxO3a are mutated from Ser or Thr to Ala (triple mutant [TM]). Therefore, nuclear localization of the FoxO3a-TM-ER fusion protein is controlled by the addition of 4-OHT, not Akt (Fig. 5A). We introduced FoxO3a-TM-ER expression into ARPE-19 retinal pigment epithelial cells that already contained a tet-on myr-Akt cassette. Of note, we also attempted to transduce human telomerase reverse transcriptase (hTERT)-immortalized fibroblasts but failed to obtain healthy cell populations after dual transduction with both myr-Akt and Fox3a-TM-ER lentivirus vectors (data not shown).

We first validated that the FoxO3a-TM-ER ARPE-19 cells were doxycycline inducible for myr-Akt expression and that the addition of 1 μM 4-OHT resulted in the nuclear localization of the FoxO3a-TM-ER fusion protein (Fig. 5B and C). Next, we induced myr-Akt expression for 24 h and then infected the cells at an MOI of 2 TCID_50_/cell in the presence or absence of 1 μM 4-OHT. We conducted these studies with two different HCMV strains: TB40/E, a strain used throughout this study up to this point, and AD169 r*UL131*, which replicates more robustly in ARPE-19 cells than TB40/E does (44, 45). In repeated experiments, the addition of 4-OHT almost entirely rescued the ~1-log replication defect observed during induction of myr-Akt (Fig. 5D and E). In three independent biological replicates with AD169 r*UL131*, the fold defect observed at 8 dpi for myr-Akt versus control (carrier-alone) conditions was 8.7-fold (range, 7.5- to 11.1-fold), but in the presence of 4-OHT, the average defect was only 1.8-fold (range, 2.8- to 1.3-fold). This difference was statistically significant (*P* = 0.0062) by an unpaired, two-tailed *t* test (Fig. 5E). The rescue of viral replication kinetics defects for both viral strains was accompanied by the reversal of defects in viral DNA synthesis and viral protein expression that were seen during myr-Akt expression in the absence of 4-OHT (Fig. 5F and G). From these experiments, we conclude that artificial nuclear localization of a FoxO3a is sufficient to reverse the replication defects that occur during expression of constitutive Akt activity.

Collectively, our results argue that FoxO family transcription factors play a critical role in the HCMV lytic replication cycle and that HCMV relies on Akt inactivation to drive FoxOs into the nucleus.

## DISCUSSION

As obligate intracellular parasites, cytolytic viruses routinely rewire host cell signaling pathways during infection, for instance, to upregulate the synthesis of macromolecules that support their replication, evade immune surveillance, and maintain cell viability while infectious progeny virions are assembled. HCMV is no exception. Although other groups have observed that Akt becomes inactivated during HCMV infection (14, 15), it has remained unclear whether cessation of Akt signaling is required for efficient viral replication. Here, we demonstrate that constitutively active Akt causes a substantial, ~10-fold viral replication defect that is accompanied by substantially reduced viral late gene expression and defects in viral DNA synthesis (Figs. 2 and 3). Our results therefore argue that inactivation of Akt is necessary for efficient viral replication. Moreover, our findings reveal that activation of FoxO3a is a critical function of Akt shutoff during HCMV infection.

Precisely how HCMV inactivates Akt is unclear but likely involves dysregulated mTORC1 activity due to the activity of UL38, a viral immediate early protein (16). UL38 binds and inactivates TSC2, a negative regulator of mTORC1 that is ordinarily inactivated by Akt (3). Hence, the observation that HCMV encodes a TSC2 inactivator already provides a clue that Akt activity is either insufficient or missing during infection. However, UL38 also activates mTORC1 by TSC2-independent mechanisms (17). In uninfected cells during excessive growth factor stimulation, mTORC1 phosphorylates the insulin receptor substrate (IRS) family proteins, such as IRS-1 at Ser422, leading to their ubiquitination and proteasomal degradation (46, 47). Because tyrosine-phosphorylated IRS proteins provide important membrane-proximal docking sites for class I PI3Ks, their degradation limits the recruitment of PI3Ks to intracellular domains of activated growth factors, thereby preventing PI3Ks from activating Akt.

Overall, even though a plausible mechanism exists to explain Akt inactivation during infection, our finding that constitutive Akt activity hinders HCMV replication is intriguing. Viruses that utilize cap-dependent mRNA translation deploy diverse mechanisms to maintain mTORC1 in an active state, in large part to stimulate high levels of mRNA translation (48, 49); in examples where mTORC1 is activated by viral factors that intercede downstream of PI3K/Akt, inactivation of Akt would likely be interpreted as a by-product of mTORC1 hyperactivation. In such cases, constitutive activation of Akt might be predicted to be of little to no consequence for the virus. Our findings from siRNA knockdown studies (Fig. 4) and experiments dislocating nuclear localization of FoxO3a from Akt (Fig. 5), however, underscore a pivotal role during HCMV infection for one Akt substrate, FoxO3a, a transcription factor whose activity is negatively regulated by Akt. Therefore, our results suggest an explanation for why constitutive Akt activity is in fact detrimental to HCMV.

Our data show that small-molecule (4-OHT)-controlled nuclear localization of FoxO3a all but entirely reverses the viral replication defects that occur in the presence of constitutive Akt activity (Fig. 5). The use of a chimeric FoxO3a-estrogen receptor fusion protein in the context of inducible myr-Akt expression provides advantages over siRNA knockdown approaches, which in our hands showed incomplete knockdown of FoxO TF, and is also arguably superior to assessing viral replication in cells deleted for individual Forkhead box O genes, since wholesale gene ablation may lead to pleiotropic effects. We previously demonstrated that FoxO3a stimulates reexpression of major immediate early (MIE) genes from intronic promoter elements in the MIE region (21), and the early defects we observed here in mRNA accumulation for IE2 and spliced MIE promoter transcripts during myr-Akt expression are consistent with a potential role for FoxO TF in transcription from the MIE locus (Fig. 3).

Even though subtle defects in expression of MIE proteins, such as IE2, may dampen the expression of a wide array of different early- and late-phase lytic cycle genes, we still cannot exclude the possibility that FoxO transcription factors play broader roles during the viral lytic cycle. One possibility is that FoxO3a is necessary to upregulate cellular stress response genes, for instance, to address the burden of oxidative stress associated with lytic replication. Another, nonmutually exclusive possibility is that HCMV lytic replication genes are directly regulated by FoxO3a, perhaps to regulate viral reactivation from latency in response to extrinsic or intrinsic stressors, much the same way that cellular genes are regulated by FoxO3a to coordinate stress responses. Additional studies will no doubt be needed to decipher how FoxO transcription factors promote efficient HCMV replication.

While our studies here focused on the viral lytic cycle, our findings are also relevant to understanding latency in HCMV and possibly a wide range of herpesviruses. Results from studies of herpes simplex virus 1 (HSV-1), murine gammaherpesvirus 68, human herpesvirus 8 (Kaposi’s sarcoma-associated herpesvirus [KSHV]), and HCMV collectively underscore a theme in which inhibition of the PI3K-Akt pathway broadly promotes herpesvirus reactivation from latency (15, 50, 51). Further, the KSHV latent protein LANA2 (vIRF3) inhibits FoxO3a activity, which may suggest a role for FoxO3a in KSHV reactivation from latency (52). The latter observation fits well with our previous findings in support of a role for FoxO3a in HCMV reactivation from latency (21, 22). Taken together, our data highlight that the PI3K-Akt-FoxO3a axis may impact dynamic infection states, such as lytic replication versus establishment of latency, as well as progression of the lytic cycle itself, in a broad range of herpesviruses.

## MATERIALS AND METHODS

### Cells and viruses.

Human telomerase reverse transcriptase (hTERT)-immortalized human foreskin fibroblasts (fibroblasts), derived from ATCC HFF-1 cells (SCRC-1041), were maintained in Dulbecco’s modified Eagle’s medium supplemented with 5% to 10% newborn calf serum (NCS) (Millipore Sigma) and antibiotics (complete DMEM), as described previously (53, 54). HEK-293T cells were purchased from GenHunter Corp. (Nashville, TN). The retinal pigment epithelial cell line ARPE-19 was purchased from the ATCC (catalog no. CRL-2302). All cells were cultured in Dulbecco's modified Eagle's medium (DMEM) (Corning; catalog no. 10013CV) supplemented with 25 μg/mL gentamicin, 10 μg/mL ciprofloxacin HCl, and either 5% fetal bovine serum (FBS) (Sigma-Aldrich [catalog no. F2442] or Gemini Biosciences, Foundation B [catalog no. 900-208]) or 5% NCS (Gemini Biosciences; catalog no. 100-504). Studies using doxycycline induction of myr-Akt or myr-Akt K179M cells were conducted using Opti-MEM medium (Gibco, Thermo Fisher) supplemented with antibiotics as described above, except that tet-approved FBS, certified free of trace levels of tetracycline (or its derivatives), (Clontech Labs; catalog no. 631106) was used as the source of FBS, at 3% final concentration.

Virus was reconstituted by electroporation of HCMV bacterial artificial chromosomes (BACs) into fibroblasts as previously described. Stocks of HCMV strain AD169rv repaired for UL131 (ADr131) (45) were prepared by infection at a low MOI on ARPE-19 cells until 100% cytopathic effect (CPE) was observed. Virus-containing culture supernatants were then subjected to centrifugation (1,000 × *g*) for 10 min to pellet cellular debris. The supernatant was then divided between 25- by 89-mm centrifuge tubes (Beckman Coulter; catalog no. 326823 or 355631), and a 7.5-mL 20% sorbitol cushion (25 mM Tris-HCl, pH 8.0, 1 mM MgCl_2_, 100 μg/mL bacitracin) was added to the bottom of each tube. The tubes were then loaded onto a SW 32 Ti swinging-bucket rotor (Beckman Coulter) and ultracentrifuged at 24,000 × *g* for 1 h at 4°C. Virion pellets were resuspended in DMEM containing 20% NCS. In the case of strain TB40/E (55) and its green fluorescent protein (GFP)-expressing derivative TB40E_5 (56), virus stocks were prepared by low-MOI infection of fibroblasts until 100% CPE was observed. Cell-associated virus was then released by Dounce homogenization of pelleted infected cells, clarified of cell debris by centrifugation (1,000 × *g*, 10 min), and combined with the cell-free supernatants before ultracentrifugation and resuspension, as described above. TB40/E virions were resuspended in 20% NCS–DMEM, except for those used in the studies shown in Fig. 1B and C, where DMEM supplemented with 1.5% bovine serum albumin (BSA) fraction V (Gibco; catalog no. 15260-037) was used for resuspension. Viral replication kinetics studies were carried out as described previously (53). TB40/E_5 (GFP) was used in lieu of standard TB40/E for the experiment shown in Fig. 2C.

### Construction of plasmids for this study.

Plasmid pLenti-X1-Hygro-mCherry 2A-3×HA FoxO3a-TM-ER was generated as follows: pLenti-X1 Hygro mCherry RAMP4 (Addgene; catalog no. 118391, a gift of Jacob Corn) was linearized with XcmI, and the 10.205-kb fragment was assembled using HiFi Assembly master mix (NEB) in a 4-way reaction together with the 2.383-kb NheI/KpnI-released FoxO3a-TM-ER fragment from plasmid HA-FoxO3a-TM-ER (Addgene; catalog no. 8353, a gift of Michael Greenberg) and two synthetic double-stranded DNA (dsDNA) IDT gBlocks, FoxO3A_ER_gBlock1 (244 bp) and FoxO3A_ER_gBlock2 (248 bp) (see Table S1 in the supplemental material). (Note that TM in FoxO3a-TM indicates “triple mutant” for all three Akt sites, T32A, S253A, S315A.) The final lentivirus vector plasmid encodes, under the control of the EF1alpha promoter, mCherry fused to a P2A “self-cleaving” peptide, followed by 3 tandem copies of the HA tag (YPYDVPDYA) linked to FoxO3a-TM, and then finally fused to the hormone binding domain (amino acids [aa] 281 to 599) of the G525R mutant murine estrogen receptor alpha (57, 58) and then terminating with a UGA stop codon.

10.1128/mbio.01042-22.1 TABLE S1 List of quantitative PCR primers and custom-synthesized nucleotides used in this study. Download Table S1, DOCX file, 0.01 MB. Copyright © 2022 Zhang et al. 2022 Zhang et al. https://creativecommons.org/licenses/by/4.0/ This content is distributed under the terms of the Creative Commons Attribution 4.0 International license.

To construct tet-on plasmids expressing either myr-Akt or myr-Akt-K179M, we digested the plasmid pECE-myrAkt Δ4-129 (Addgene; catalog no.10841, a gift of Richard A. Roth) with EcoRI-HF and NotI. (Note that Δ4-129 signifies deletion of the Akt pleckstrin homology domain.) T4 DNA ligase was then used to insert the released myr-Akt fragment into an agarose gel-purified backbone of the all-in-one tet-on lentiviral vector plasmid pOUPc_UL148^HA^ (59), which had been opened using EcoRI-HF and NotI to release the *UL148^HA^* insert. This resulted in pOUPc-myrAkt (Δ4-129). A K179M version, pOUPc-K179M myrAkt (Δ4-129), was generated by first double-digesting pECE-myrAkt (Δ4-129) using EcoRI-HF and XbaI to release the 1,199-bp fragment containing myr-Akt. This fragment was ligated into the pSP72 vector (Promega) by using T4 DNA ligase using the EcoRI and XbaI site on its polylinker, resulting in pSP72-myrAkt (Δ4-129). Then, pLNCX myr-Akt_K179M (Addgene; catalog no. 9906, a gift of William R. Sellers) was digested with SmaI plus DraIII to release a 289-bp fragment containing the K179M mutation in the context of *Akt1*. The 289-bp fragment was then inserted into a SmaI-plus-DraIII-linearized pSP72-myrAkt (Δ4-129) backbone using T4 DNA ligase, yielding pSP72-K179M-myrAkt (Δ4-129). Finally, pSP72 K179 myrAkt (Δ4-129) and pOUPc-myrAkt (Δ4-129) were separately double digested with EcoRI and BlpI. After agarose gel purification, the 930-bp fragment from pSP72 K179 myrAkt (Δ4-129) containing the K179M mutation was ligated into the 12,341-bp fragment generated after BlpI plus EcoRI double digestion of pOUPc-myrAkt (Δ4-129). All plasmids were confirmed by Sanger sequencing at Genewiz, LLC. (Piscataway, NJ).

### Lentivirus vector transduction.

All lentivirus vector particles were generated in HEK-293T cells by cotransfection with plasmids pMD2.g and psPAX2 (Addgene; catalog no. 12259 and12260, gift of Didier Trono), exactly as described previously (59), except that PEI MAX (Polyfect) transfection reagent was used instead of TransIT-293 reagent. To generate cells expressing mCherry_3×HA_FoxO3a_TM_ER, ARPE-19 epithelial cells were transduced as described previously (59) and selected in complete DMEM using 50 μg hygromycin B for three passages. These cells were then transduced with the tet-on lentiviral vector pOUPc-myr-Akt (ΔPH domain, aa 4 to 129). The cells were further selected with hygromycin (50 μg/mL) and 2 μg/mL puromycin for three passages and then subjected to fluorescence-activated cell sorting on a FACSAria III (BD Biosciences) to enrich for mCherry-expressing cells. mCherry-positive cells were expanded in DMEM containing 10% FBS. During the cell expansion process, selective antibiotics were maintained at 50 μg/mL hygromycin and 2 μg/mL puromycin. Doubly transduced cells were validated by adding 1 μM 4-OHT or ethanol carrier-alone control for 1 h and then fixed for induced nuclear localization of the hemagglutinin (HA) staining signal using rabbit anti-HA (Bethyl; A190-108A), with goat anti-rabbit Alexa Fluor 488 (Invitrogen; A11008) for secondary detection (see “Confocal microscopy and immunofluorescence” below for details).

### Western blotting.

For Western blotting experiments, cells were seeded into 6-well cluster plates at 1 × 10^6^ cells per well and infected with HCMV at an MOI of 2 TCID_50_/cell. For studies of Akt phosphorylation kinetics (Fig. 1B and C), infections using HCMV strain TB40/E were carried out in fibroblasts that had been seeded 1 day earlier at 0.5 × 10^6^ cells per well in DMEM supplemented with 5% NCS. Infections were carried out using ultracentrifuge-concentrated virus stocks that had been suspended in DMEM–1.5% BSA and diluted into fresh 5% NCS–DMEM prior to infection of cells. Infections were incubated at 37°C, in 5% CO_2_, in a humidified incubator until harvest at the indicated times postinfection. For mock infection controls, fresh medium (5% NCS–DMEM) was applied in lieu of virus-containing inoculum, and cells were returned to 1 h of incubation at 37°C, in 5% CO_2_, prior to lysis. For the experiments shown in Fig. 1B and C, samples were harvested by aspirating the medium from the wells, washing in ice-cold Dulbecco’s phosphate-buffered saline (Genesee Scientific; catalog no. 25-508), and then lysing in 0.2 mL 1× Laemmli buffer (2% sodium dodecyl sulfate [SDS], 0.25% bromophenol blue, 10% glycerol) supplemented with 1× protease and phosphatase inhibitor cocktails (APExBio, Houston, TX; catalog no. K1007 and K1012, respectively) and 5% (vol/vol) β-mercaptoethanol. Lysates were transferred to microcentrifuge tubes and stored frozen until further processing by SDS-PAGE, at which time the samples were denatured at 65°C for 10 min and then vortexed for 1 min at maximum speed prior to loading 30 μL per lane on a 10-lane Bio-Rad Mini-PROTEAN 3 minigel (10% acrylamide).

For all other immunoblot experiments, fibroblasts or ARPE-19 epithelial cells were treated, where indicated, with doxycycline (Dox) at 100 ng/μL or carrier alone for 24 h prior to infection, while for 4-OHT (1 μM), drug was added immediately after infection. Drug treatments (and carrier controls) were replenished every 24 h with fresh medium matching the other conditions (e.g., with or without Dox or with or without 4-OHT). The cells were lysed at the indicated time points for analysis by Western blotting, as described previously (60). Briefly, after aspiration of the medium and washing the cells in ice-cold PBS, 300 μL radioimmunoprecipitation assay (RIPA) buffer (50 mM Tris [pH 7.4], 150 mM NaCl, 0.1% sodium dodecyl sulfate [SDS], 0.5% sodium deoxycholate, 1% NP-40), supplemented with 1× protease cocktail, EDTA-free (APeXBIO; catalog no. K1010), and 1× phosphatase inhibitor cocktails 1 and 2 (APeXBIO; catalog no. K1012-10 and K1013-10), was applied per well and cells were subjected to scraping with a 1,000-μL pipette tip. Lysates were then transferred to microcentrifuge tubes and stored at −70°C until analysis. To process frozen lysates for SDS-PAGE, samples were thawed and then centrifuged (18,000 × *g*) for 30 min at 4°C to remove cellular debris. Protein concentrations of supernatants were then determined using the bicinchoninic acid (BCA) assay (Pierce BCA protein assay kit; catalog no. 23225) according to the manufacturer's instructions and then normalized to match the least-concentrated sample. Normalized lysates were mixed 3:1 with 4× Laemmli buffer containing 20% β-mercaptoethanol, heated at 85°C for 5 min, and then spun down briefly at room temperature. After proteins were resolved on 10% acrylamide (29:1 acrylamide:bis-acrylamide) SDS-PAGE gels in 25 mM Tris-Cl–250 mM glycine–0.1% SDS, proteins were transferred overnight to nitrocellulose membranes (Whatman Protran; 0.45-μm pore size) at 4°C using a Bio-Rad Criterion blotter with standard buffer (25 mM Tris, 192 mM glycine, pH 8.3, 20% methanol buffer) and the current was set to 150 mA.

After transfer, nitrocellulose membranes were blocked for 1 h at room temperature in 5% BSA (Thermo Fisher; catalog no. BP1605-100) in PBS for the results shown in Fig. 1B and C, and for all other Western blot results, membranes were blocked in PBS containing 0.1% Tween 20 and 5% nonfat powdered milk (LabScientific; catalog no. M0842) (5% PM–PBST). For Western blot results shown in Fig. 1B and C, primary and secondary antibodies were each incubated for 1 h at room temperature in PBS supplemented with Tween 20 at 0.1%. For all other Western blots, primary antibody was incubated overnight in 5% PM–PBST at 4°C, and secondary antibody was incubated at room temperature in 5% PM–PBST for 1 h. IE2 was detected using mouse monoclonal antibody (MAb) clone 5A8.2 (Millipore, Inc.; catalog no. MAB8140), and GAPDH was detected using mouse MAb 60004-1-Ig (Proteintech). All other viral proteins and the HA epitope tag were detected as described previously (53, 60). Details on all antibodies used in this study are provided in Table S2 in the supplemental material. Western blot results were captured using a LI-COR Odyssey system (LI-COR, Inc., Lincoln, NE). Image Studio 5.25 software (LI-COR) was used to quantify signals from far-red fluorescent dye-labeled secondary antibodies.

10.1128/mbio.01042-22.2 TABLE S2 List of antibodies used in this study. Download Table S2, DOCX file, 0.02 MB. Copyright © 2022 Zhang et al. 2022 Zhang et al. https://creativecommons.org/licenses/by/4.0/ This content is distributed under the terms of the Creative Commons Attribution 4.0 International license.

### RT-qPCR.

For quantification of viral RNA, total RNA was isolated from infected cells using an RNeasy minikit with an on-column DNase digestion step, per the manufacturer's instructions (Qiagen, Inc., Valencia, CA). For each sample, 1,000 ng total RNA was used as a template to produce oligo(dT)-primed cDNA by use of a qScript cDNA synthesis kit (Quanta Bioscience, Gaithersburg, MD). The resulting cDNAs and control samples without reverse transcriptase (RT) were diluted 5-fold with water and used as a template in qPCRs to quantify the abundance of the transcripts of the indicated viral genes and of cellular GAPDH (glyceraldehyde-3-phosphate dehydrogenase) transcripts, which were used for normalization. cDNA was then used in a standard SYBR green real-time PCR with NEB Luna master mix (M3003E) to measure gene expression using the indicated primer pairs (Table S1), as described previously (59). Reaction conditions were 95°C for 15 s and 60°C for 1 min, repeated for 40 cycles, with a 10-min hot start at 95°C. Relative mRNA levels were quantified using the 2^−ΔΔ^*^CT^* method, with standardization to RT-qPCR results for *GAPDH*. Control samples in which reverse transcriptase was intentionally left out during the cDNA synthesis step uniformly produced qPCR results indistinguishable from the results of RT-qPCR on RNA samples from mock-infected cells (not shown).

### siRNA reverse transfection.

siRNAs were reverse transfected into hTERT-immortalized human foreskin fibroblasts (HFFs) using Lipofectamine RNAiMAX reagent (Thermo Fisher) according to the manufacturer’s instructions. Briefly, two mixes were prepared separately. Mix 1 was prepared by adding 30 pmol of siRNA to 150 μL nonsupplemented Opti-MEM medium (Thermo Fisher) and then gently mixing. Mix 2 was prepared by adding 9 μL of RNAiMAX reagent to 150 μL nonsupplemented Opti-MEM medium. Mixes 1 and 2 were then combined, immediately transferred to an empty well of a 6-well plate, and incubated at room temperature for 5 min. Approximately one million cells were then added to the well in 2.0 mL of DMEM containing 5% NCS (10 nM siRNA, final concentration). At 24 h later, the cells were infected at an MOI of 1 TCID_50_ per cell with HCMV strain TB40/E. At 2 h after infection, the cells were washed twice in PBS (137 mM NaCl, 2.7 mM KCl, 10 mM Na_2_HPO_4_, and 1.8 mM KH_2_PO_4_, pH 7.4), allowing the PBS to sit on the cells for 5 min each time, and then fresh complete DMEM (5% NCS) was added. At the indicated time points, supernatants were collected for viral titer determination. siRNAs used in this study were as follows: Dharmacon nontargeting control pool 2 (catalog no. D-001206-14-05), SignalSilence FoxO1 siRNA I (Cell Signaling Technology; catalog no. 6242), and SignalSilence FoxO3a siRNA I (Cell Signaling Technology; catalog no. 6302). RNA was extracted from HFFs at the indicated time points using the RNeasy minikit (Qiagen, Hilden, Germany).

### Induction of FoxO-TM-ER fusion protein nuclear localization.

For the experiment shown in Fig. 5, ARPE-19 cells were seeded at 1 × 10^5^ cells per well in 24-well cluster plates. Where indicated, cells were treated with doxycycline (100 ng/mL) for 24 h and then with (Z)-4-hydroxytamoxifen (4-OHT; Sigma-Aldrich catalog no. 508225) at a final concentration of 1 μM for 1 h to induce myr-Akt or nuclear localization of FoxO3a-TM-ER, respectively. Each drug was applied from a 1,000× stock solution (in water for doxycycline and in ethanol for 4-OHT); therefore, carrier-alone control wells were treated with the same volume of absolute ethanol and/or water (0.1%, final concentration). Next, TB40/E or AD169 *rUL131* virus was applied at an MOI of 2 (2 TCID_50_ per cell), as indicated. For viral replication kinetics studies, infected cell supernatants were collected at the indicated time points and stored at −80°C until determination of titer by the TCID_50_ assay using the Reed and Muench method, as described elsewhere (53).

### Viral DNA synthesis assays.

Total DNA was isolated from infected cells using the DNeasy blood and tissue minikit (Qiagen; catalog no. 69506). Briefly, cells were seeded into 24-well plates as described above for replication kinetics studies, and at the indicated times postinfection, culture medium was removed and cells were washed three times with PBS, allowing PBS to sit on cells for 5 min per wash. After the PBS wash was aspirated, 200 μL Qiagen buffer AL was added to each well, followed by 200 μL PBS and 20 μL of the proteinase K solution included with the kit. The cells were then scraped from the wells using a 1,000-μL-capacity pipette tip to mix them into the lysis buffer solution. Lysates were then transferred to 1.5-mL microcentrifuge tubes and processed per the manufacturer’s instructions to isolate total DNA. Absolute quantification of viral DNA copies with specific primers targeting the viral *UL69* gene was performed using quantitative PCR (qPCR) alongside duplicate standard curves of serial 10-fold dilutions of TB40/E BAC DNA, from 10^7^ to 10^2^ copies per well, for determination of viral copy number, using the same qPCR reagents described above (see “RT-qPCR”). Meanwhile, a separate standard curve was generated using a plasmid containing a fragment of the human 18S ribosomal DNA (rDNA) gene, from 10^7^ to 10^2^ copies per well, to allow absolute quantification of 18S rDNA copies. Viral *UL69* copies were then normalized across samples to cellular 18S rDNA copies (Table S1).

### Confocal microscopy and immunofluorescence.

Confocal immunofluorescence was carried out essentially as described previously (60). For the experiment shown in Fig. 1, hTERT-immortalized HFFs were seeded on a 12-mm, circular, number 1 thickness microscope cover glass (200121; Azer Scientific, Morgantown, PA) and infected with HCMV strain TB40/E at an MOI of 1 at 37°C in a humidified 5% CO_2_ incubator. At 2 h later, culture medium was removed, and cells were washed twice in PBS and then incubated in fresh complete medium containing 5% NCS. The cells were then fixed at the indicated times postinfection by removing the culture medium, washing with PBS, and then applying a solution of 4% paraformaldehyde (PFA) in PBS for a minimum of 20 min at room temperature. After fixation, PFA was removed and cells were washed three times in PBS, allowing 5 min for each wash. The cells were then permeabilized for 10 min using 0.1% Triton X-100 (in PBS) at room temperature and then washed an additional three times in PBS. After washing, cells were blocked at 37°C for 45 min in a solution of 5% normal goat serum (Rockland Immunochemicals) in PBS, washed another three times in PBS, and then additionally blocked using 1% human BD Fc block (BD Biosciences) in PBS at 37°C for an additional 45 min. The cells were then washed again three times in PBS, for 5 min per wash, and then incubated with antibodies specific for IE1 and FoxO3a (Cell Signaling Technology) at 4°C overnight (Table S2). The next day, thecells were washed in PBST (PBS containing 0.1% Tween 20). After three washes in PBST, the cells were incubated with Alexa Fluor 488-conjugated goat anti-rabbit and Alexa Fluor 405-conjugated goat anti-mouse secondary antibodies (Invitrogen). After secondary antibody incubation, the cells were washed three times with PBST, 5 min per wash. Next, Prolong antifade mounting medium without DAPI (4′,6-diamidino-2-phenylindole) (Thermo Fisher) was applied to a slide, and the cover slip was inverted onto the mounting medium and sealed with nail polish around the edge of the coverslip. Confocal imaging was obtained on a Leica TCS SP5 confocal microscope using a 63× oil immersion lens (Leica Microsystems). In Fig. 5C, the same staining procedure was used except that the ARPE-19 cells were not infected, and anti-HA antibody (Bethyl), followed by Alexa 488-conjugated goat anti-rabbit secondary antibody, was used to detect FoxO3a-TM-ER by virtue of its 3×HA epitope tag. Finally, nuclei were counterstained using a 10-min incubation in PBS containing 10 μg/mL Hoechst 33342 (Millipore Sigma; catalog no. 14533). The cells were then washed three times in PBS and mounted in Prolong antifade mounting medium without DAPI.

### Statistical analysis.

Where indicated, statistical analyses were carried out using Prism 9 for MacOS (version 9.3.1).
