# Supplementary figures and images for: The Akt Forkhead Box O Transcription Factor Axis Regulates Human Cytomegalovirus Replication
Source: mBio. 2022 Aug 10;13(4):e01042-22. doi: 10.1128/mbio.01042-22 (PMC9426471; doi:10.1128/mbio.01042-22)

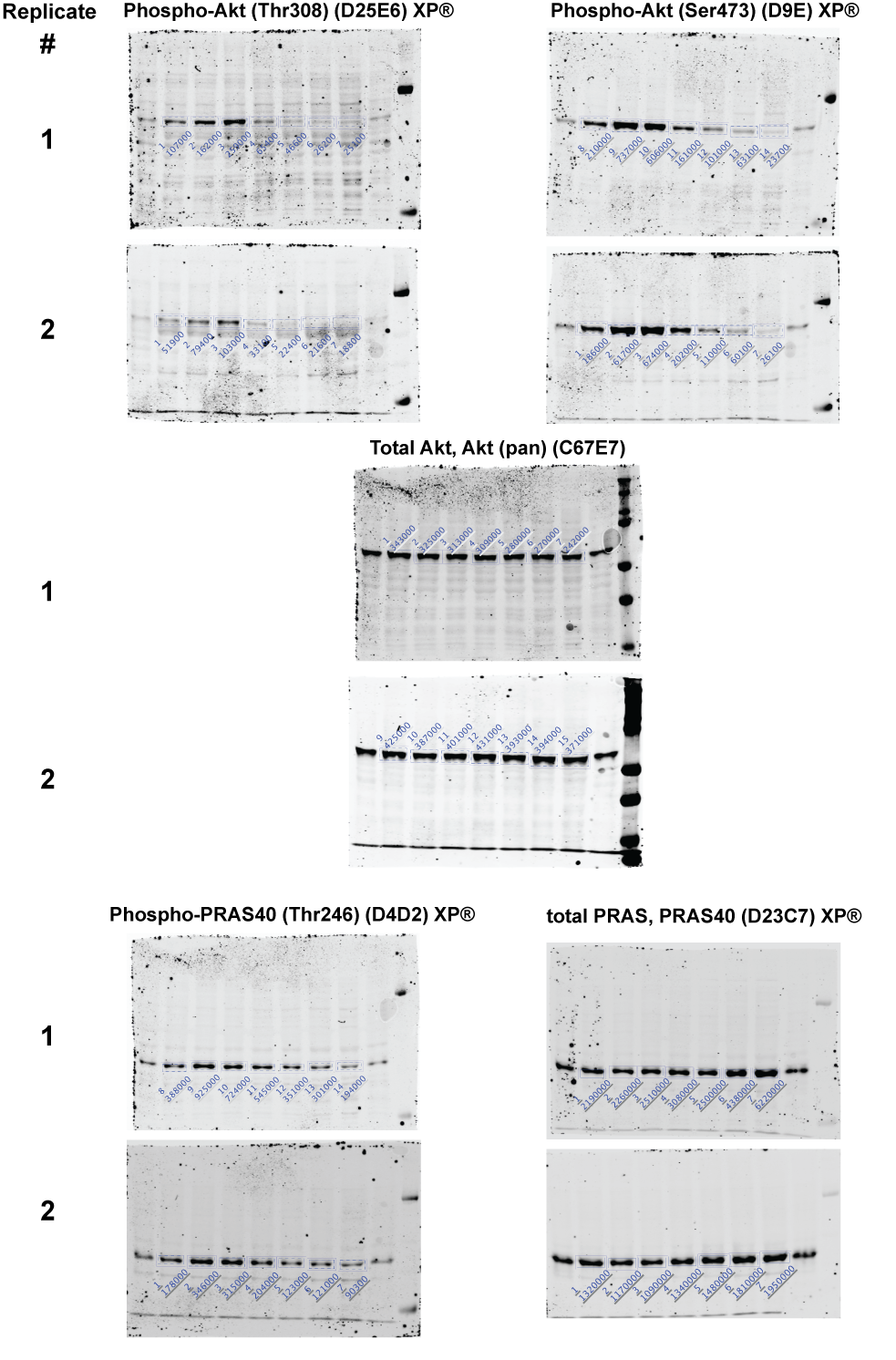

Supplement: FIG S1 [file mbio.01042-22-s0001.tif]
