# Supplementary material for: The Akt Forkhead Box O Transcription Factor Axis Regulates Human Cytomegalovirus Replication
Source: mBio. 2022 Aug 10;13(4):e01042-22. doi: 10.1128/mbio.01042-22 (PMC9426471; doi:10.1128/mbio.01042-22)

## Extended Data for Fig 1D; includes mock infection series

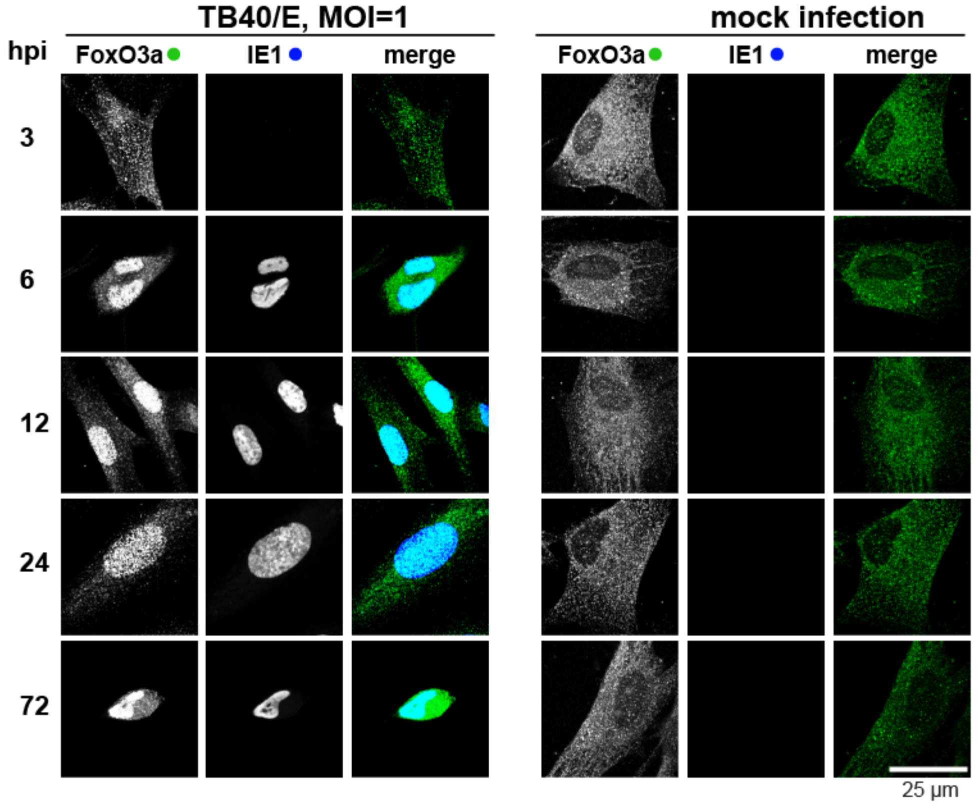


## Extended Data for Fig 2D; full time course (TB40/E, MOI=1)

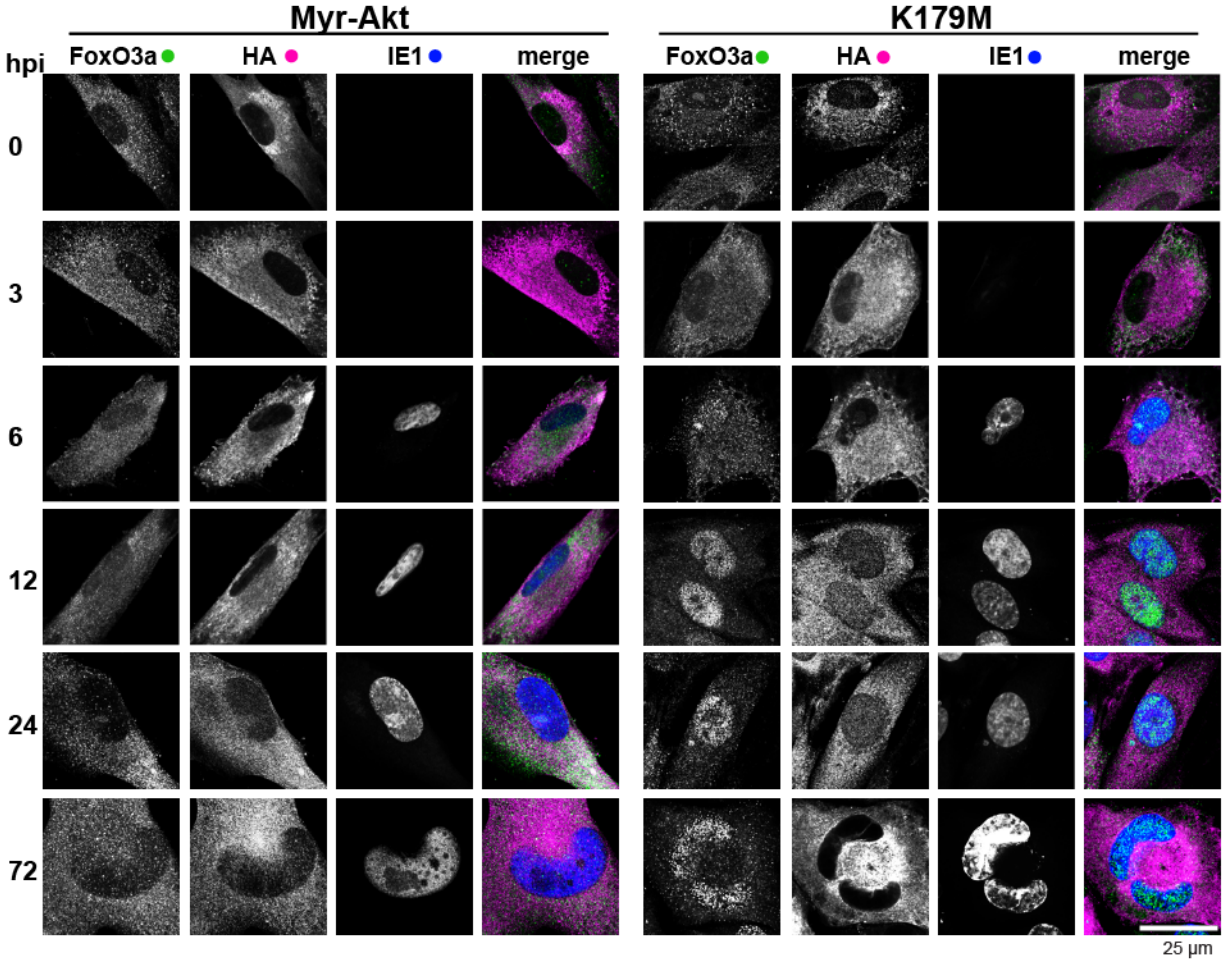

Supplement: FIG S2 [file mbio.01042-22-s0002.pdf]
