# Supplementary material for: The Akt Forkhead Box O Transcription Factor Axis Regulates Human Cytomegalovirus Replication
Source: mBio. 2022 Aug 10;13(4):e01042-22. doi: 10.1128/mbio.01042-22 (PMC9426471; doi:10.1128/mbio.01042-22)
Supplement: FIG S3 [file mbio.01042-22-s0003.pdf]

SI Figure S3, Quantification of Western blot experiments shown in Fig 4D-4E

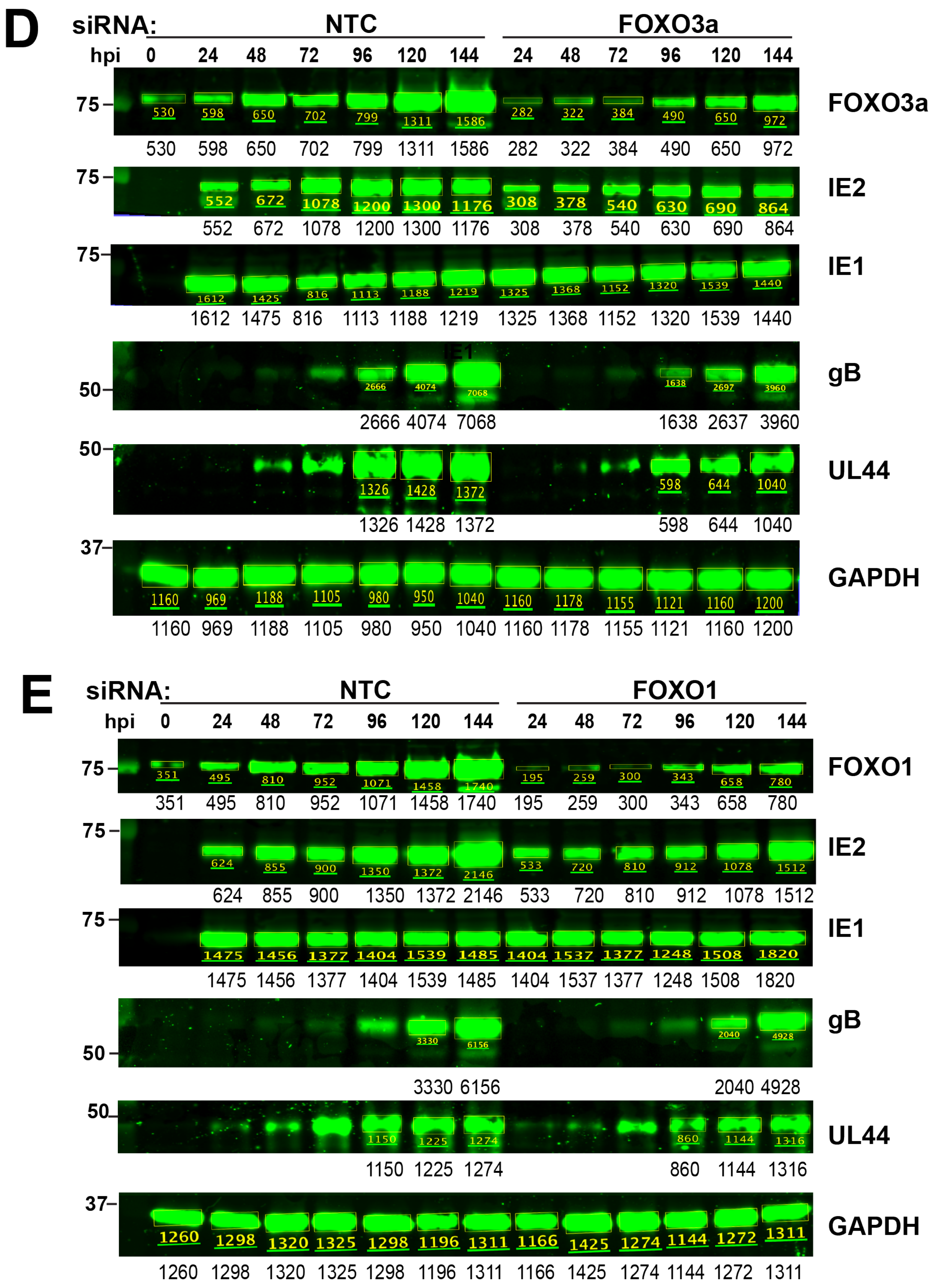

Li-Cor Image Studio version 5.2.5 (for MacOS) was used to quantify signal from indicated bands, using median background subtraction mode. Briefly, rectanges (as shown) were manually drawn around the indicated bands, and the software generates a measurement of the signal within each rectangle. IRDye 800CW conjugated antibodies were used for quantifications shown here, so the 800 nm channel was used to generate the intensity measurements. Measurements are shown in yellow underneath each band that was measured, and again, in black text below each Western blot panel. Data here are for quantification of Western blots shown in Fig 4D and 4E.
