# Supplementary material for: The Akt Forkhead Box O Transcription Factor Axis Regulates Human Cytomegalovirus Replication
Source: mBio. 2022 Aug 10;13(4):e01042-22. doi: 10.1128/mbio.01042-22 (PMC9426471; doi:10.1128/mbio.01042-22)
Supplement: TABLE S1 [file mbio.01042-22-s0004.docx]

**Supplemental Table S1. qPCR primers and synthetic custom deoxynucleotide sequences used in this study.**

| **Name** | **Sequence (5'-to-3')** |
| --- | --- |
| IRS1_FWD | GTAACGTGAGACGGAGCATATAG |
| IRS1_REV | ACGCTGTGGTTTGGAGATT |
| iP1-derived_FWD | CTTAAGGCAGCGGCAGAA |
| iP1-derived_REV | CAAGGACGGTGACTGACTC |
| iP2-derived_FWD | TAGCTGACAGACTAACAGAC |
| iP2-derived_REV | AGGACTCCATCGTGTCAAGG |
| IE1 (UL123)_FWD | TGACCGAGGATTGCAACGA |
| IE1 (UL123)_REV | CCTTGATTCTATGCCGCACC |
| IE2 (UL122)_FWD | CAGAACTCGGTGACATCCT |
| IE2 (UL122)_REV | CCGGTGCTACTGGAATCG |
| MIEP_Spliced_FWD | AGAGTGACTCACCGTCCTT |
| MIEP_Spliced_REV | GTCAGGGTCCATCTTTCTCTTG |
| UL38_FWD | TCTGTTCGCAAGGTGCTTAC |
| UL38_REV | GATGAGCACGATGAGTTGGT |
| UL69_FWD | CGTCCAGTTCGTCGTCAATAA |
| UL69_REV | CCTACGACTTTCGGTTCTTCTC |
| GAPDH_FWD | CTGTTGCTGTAGCCAAATTCGT |
| GAPDH_REV | ACCCACTCCTCCACCTTTGAC |
| FoxO3a_ER_gBlock_1 | AGTACGAACGCGCCGAGGGCCGCCACTCCACAGGGGGTATGGACGAACTGTATAAAGGCTCAGGGGCTACAAATTTTAGTCTCCTTAAACAAGCCGGAGATGTCGAAGAGAACCCTGGGCCTTACCCATATGACGTTCCTGACTATGCCGGTTACCCCTATGATGTGCCAGATTATGCCGGATCTTATCCTTACGATGTACCTGACTATGCTAGCCTCCCGGGGAATTCGGCAGAGGCACCGGC |
| FoxO3a_ER_gBlock_2 | ﻿TCTCTACAACATGAAATGCAAGAACGTGGTACCCCTGTATGACCTGCTCTTGGAAATGTTGGATGCACATCGACTCCACGCCCCAGCTAGTAGAATGGGTGTCCCTCCGGAGGAGCCATCACAGACCCAGCTGGCCACCACCAGCTCCACTTCAGCACATTCCTTACAAACCTACTACATACCCCCGGAAGCAGAGGGCTTCCCCAACACGATCTGAACGGCATGGACGAGCTGTACAAGGGTGGATC |
