# Supplementary material for: The Akt Forkhead Box O Transcription Factor Axis Regulates Human Cytomegalovirus Replication
Source: mBio. 2022 Aug 10;13(4):e01042-22. doi: 10.1128/mbio.01042-22 (PMC9426471; doi:10.1128/mbio.01042-22)
Supplement: TABLE S2 [file mbio.01042-22-s0005.docx]

**SUPPLEMENTAL TABLE S2. Antibodies used in this study**

| **Primary Antibody** | **Host species** | **Isotype** | **Clone** | **Source** | **Catalog No.** | **Dilution used** |
| --- | --- | --- | --- | --- | --- | --- |
| HA epitope | Chicken | IgY |  | Bethyl | A190-106A | 1:200 |
| HCMV major immediate-early (IE) protein IE1-72kD | Mouse | Unknown IgG | 1B12 | Thomas Shenk | n/a | 1: 50 |
| HCMV major immediate-early (IE) protein IE2-86kD | Mouse | IgG1 | 5A8.2 | Millipore, Inc. | MAB8410 | 1:1000 |
| HA epitope | Rabbit | pAb |  | Bethyl, Inc. | A190-108A | 1:1000 |
| FoxO3a (D19A7) | Rabbit | mAb |  | Cell Signaling Technology | 12829S | 1:200 |
| FoxO1 (C29H4) | Rabbit | mAb |  | Cell Signaling Technology | 2880S | 1:1000 |
| Akt (pan) (C67E7) | Rabbit | mAb |  | Cell Signaling Technology | 4691S | 1:1000 |
| PRAS40 (D23C7) XP^®^ | Rabbit | mAb |  | Cell Signaling Technology | 2691S | 1:1000 |
| Phospho-Akt (Thr308) (D25E6) XP^®^ | Rabbit | mAb |  | Cell Signaling Technology | 13038S | 1:1000 |
| Phospho-Akt (Ser473) (D9E) XP® | Rabbit | mAb |  | Cell Signaling Technology | 4060S | 1:1000 |
| Phospho-PRAS40 (Thr246) (D4D2) XP^®^ | Rabbit | mAb |  | Cell Signaling Technology | 13175S | 1:1000 |
| gB | Mouse | mAb | 27-180 | William J. Britt | n/a | 1:1000 |
| UL44 | Mouse | IgG1 | 10D8 | Virusys, Inc. | CA006-100 | 1:1000 |
| GAPDH | Mouse | IgG2b |  | Proteintech | 60004-1-Ig | 1:20,000 |
| **Secondary Antibody** | **Host species** | **Isotype** |  | **Vendor** | **Catalog No.** | **Dilution used** |
| Alexa Fluor 488 anti-Mouse IgG (H+L), cross adsorbed | Goat | IgG |  | ThermoFisher / Invitrogen | A11001 | 1:1000 |
| Alexa Fluor 488 anti-Rabbit IgG (H+L) Cross-Adsorbed | Goat | IgG |  | ThermoFisher / Invitrogen | A11008 | 1:1000 |
| Alexa Fluor 594 anti-Rabbit IgG (H+L) Cross-Adsorbed | Goat | IgG |  | ThermoFisher / Invitrogen | A11012 | 1:1000 |
| Alexa Fluor 594 anti-Mouse IgG (H+L) Cross-Adsorbed | Goat | IgG |  | ThermoFisher / Invitrogen | A11005 | 1:1000 |
| Alexa Fluor 647 anti-Chicken IgY (H+L) | Goat | IgG |  | ThermoFisher / Invitrogen | A11039 | 1:1000 |
